# Role of multimodality imaging pre-access for planning of surgical creation of arteriovenous fistulas and arteriovenous grafts in the chronic kidney disease and end-stage renal disease population

**DOI:** 10.1007/s10554-025-03356-3

**Published:** 2025-04-24

**Authors:** Abdullah Khan, Daniel Raskin, Sasan Partovi, Lee Kirksey

**Affiliations:** 1 Department of Vascular Surgery, Heart Vascular and Thoracic Institute, Cleveland Clinic, 9500 Euclid Ave, F30, Cleveland, OH 44195 USA; 2 Department of Interventional Radiology, Imaging Institute, Cleveland Clinic, Cleveland, OH USA

**Keywords:** Pre-access imaging, Dialysis access, Duplex ultrasound, Digital subtraction angiography, Digital subtraction venography, Magnetic resonance angiography, Computed tomography angiography

## Abstract

This review explores a range of imaging techniques used in the pre-surgical planning of vascular access, including duplex ultrasound (DUS), digital subtraction angiography (DSA), digital subtraction venography (DSV), CO2 Venography, magnetic resonance angiography (MRA), computed tomography angiography (CTA), and Intravascular ultrasound (IVUS). For each modality, we analyze its technical background, applications, advantages and disadvantages, and comparisons with alternative imaging options. DUS is the most widely used imaging modality in pre-surgical planning due to its low cost, non-invasiveness, absence of ionizing radiation and nephrotoxic contrast agents, and comparable accuracy in pre-access mapping with other methods. DSA and DSV have high sensitivity and specificity to visualize the arterial and venous system and are recommended when central vascular stenosis is suspected, or a simultaneous intervention is anticipated. However, their use is limited due to exposure to contrast agents and ionizing radiation. CO2-based contrast agents provide an alternative for end-stage renal disease (ESRD) patients to preserve residual renal function. MRA provides a noninvasive option with no radiation exposure and superior image resolution, yet the high cost and limited availability restrict their widespread clinical use. CTA, with its short acquisition time and high-resolution imaging, is a vital modality in intricate cases. However, radiation and contrast exposure can pose challenges in this patient population. The newer IVUS modality has a superior ability to central venous outflow obstruction compared to DSA and provides more information regarding vascular geometry and anatomy. Each imaging modality has its unique advantages and disadvantages in this patient cohort. The decision to use a particular imaging must be made on a case-to-case basis. However, following KDOQI guidelines, a combination of a patient’s medical history, physical examination, and DUS is a widely accepted standard practice in pre-surgical vascular access planning, with other imaging modalities reserved for selected patients.

## Introduction

More than 2 million people worldwide require Renal replacement therapy (RRT) for the management of end-stage renal disease (ESRD) [1]. Functioning hemodialysis access is a life-sustaining requirement for ESRD patients. Vascular access (VA) can serve either as a bridge to renal transplant in a minority of ESRD patients or as a long-term solution in non-transplant candidates. A functional VA with 300–400 ml ⁄ minute blood extraction ability is required for successful hemodialysis [2]. Hemodialysis can be achieved by three main types of VA: autogenous arteriovenous fistula (AVFs), arteriovenous grafts (AVGs), and central venous catheters (CVCs).

Autogenous AVF for chronic hemodialysis is superior to arteriovenous grafts and CVC secondary to a markedly reduced risk of mortality, infection, hematoma, bleeding, pseudoaneurysm, steal syndrome, and unplanned hospital readmissions [3, 4]. With regard to primary and secondary circuit patency, the comparative superiority of AVF versus AVG has been widely described in the literature [3, 4]. However, more contemporary literature has called this observation into question, especially for certain patient cohorts, e.g., the elderly and those with large body habitus [5–7]. AVF is the preferred choice, with AVG used when vessels are deemed unsuitable for AVF formation, and CVC is considered a temporary or last resort option [8]. However, some patients may require long-term CVC use because of difficulty in establishing a functioning permanent access, and patient preference [9]. Hence, the decision on the type of access must be tailored to individual patients. The AVF and AVF are surgically created anastomoses between a peripheral artery and vein, either directly or through a graft. The anastomosis allows increased blood pressure into the venous segment and induces venous vascular expansion and remodeling. This phenomenon is known as maturation, and it is critical for long-term success when using the access for hemodialysis.

To obtain a functional vascular access circuit, an individualized patient-centered approach should be pursued for pre-access planning, including history, physical examination, and multimodality imaging for assessment of vessel status. Arterial and venous diameter, vessel wall quality, and anatomic vessel aberrancy are independent predictors of access patency, maturation, and functional outcomes. The known impact of these key anatomic variables underscores the importance of pre-procedural imaging to improve the reliability of access circuit maturation and patency [10]. Moreover, access circuit imaging also plays a vital role in post-procedural surveillance to maintain the adequacy of circuit performance. Each imaging modality has a distinct set of advantages and disadvantages, and the choice of imaging modality needs to be individualized. Additionally, cost-effective strategies of surveillance with the prudent use of resources are necessary to efficiently conduct surveillance and mitigate harm to patients that might stem from clinically irrelevant findings seen on imaging, particularly related to surveillance ultrasound. This publication will review different imaging modalities and their uses in the dialysis circuit vascular access creation, thereby exploring the advantages and disadvantages of the different imaging modalities.

## Duplex ultrasound (DUS)

### Technical background

DUS has become an integral part of diagnostic imaging since its introduction in medicine in the 1970s [11]. Real-time B mode imaging, quickly followed by the introduction of color Doppler in 1978, enabled vascular visualization without ionizing radiation, leading to the widespread use of Doppler as a non-invasive diagnostic technique for vascular diseases [12]. DUS is now considered the initial imaging modality of choice for various vascular pathologies. Color DUS has been a reliable, non-invasive diagnostic assessment tool for vascular access circuit (VAC) planning and assessment of morphology and function [13].

### Clinical applications of the modality

DUS is a viable non-invasive imaging technique used to evaluate vessels prior to VAC and is a good predictor of circuit patency [14]. Hossain et al. compared patients who underwent pre-access DUS and those with no pre-access imaging and reported the DUS group to have lower primary failure rates (18% vs. 47%, *p* <.001), increased rates of new fistula creation (31% vs. 9%, *p* <.001), reduced fistula abandonment (39% vs. 66%; *P* <.001) and higher secondary patency rates (73% vs. 57%, p 0.01). The preprocedural use of the DUS examination also improved the time to functional fistula maturation [15] A meta-analysis of randomized control trials (RCT) and cohort studies comparing pre-access DUS to no pre-access imaging reported lower primary AVF failure and higher 1-year primary patency rates when pursuing DUS imaging prior to VAC [16].

**Fig. 1:**
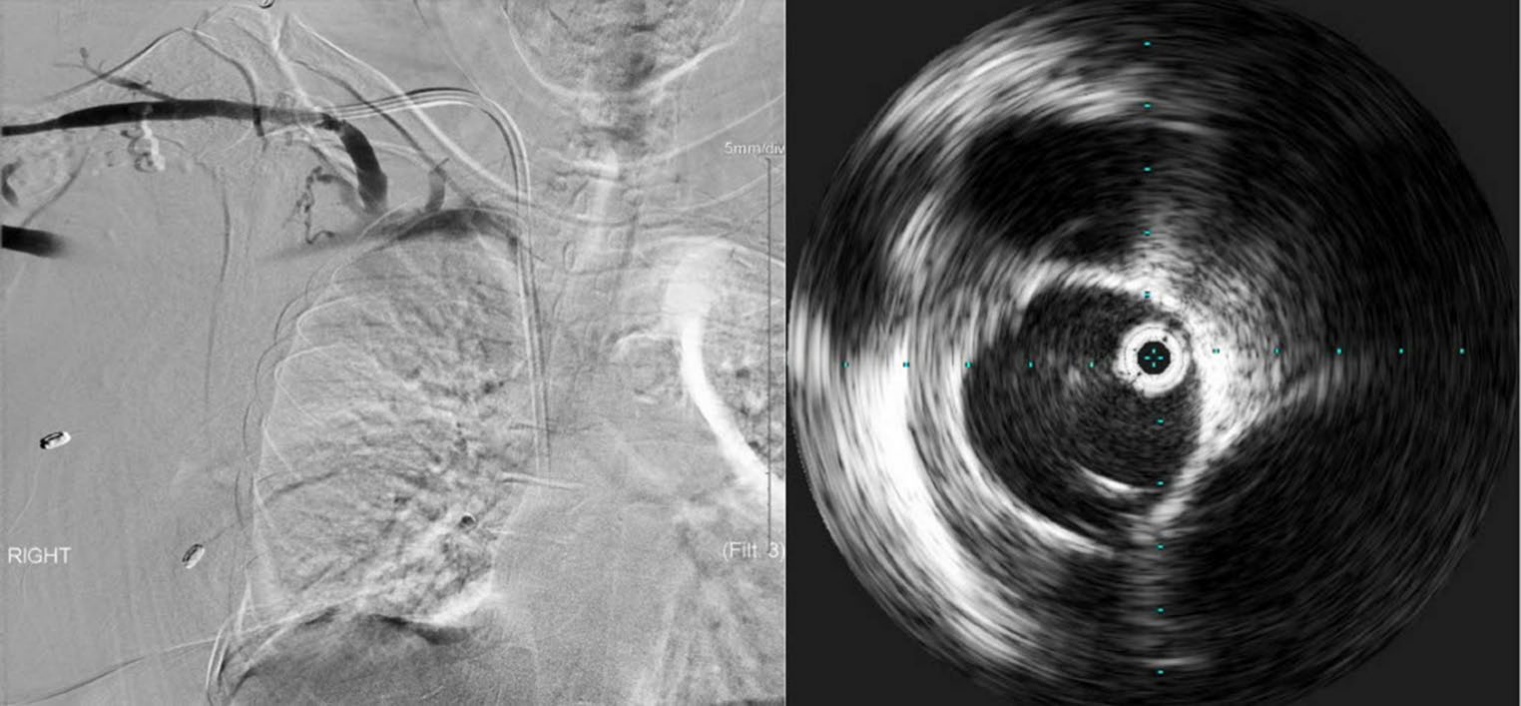
*Left Image*: Contrast Venography showing a patent right subclavian vein with no evidence of stenosis. *Right Image*: Intra-vascular Ultrasound showing the mildly diseased innominate vein with chronic echogenic wall adherent thrombotic disease

**Fig. 2:**
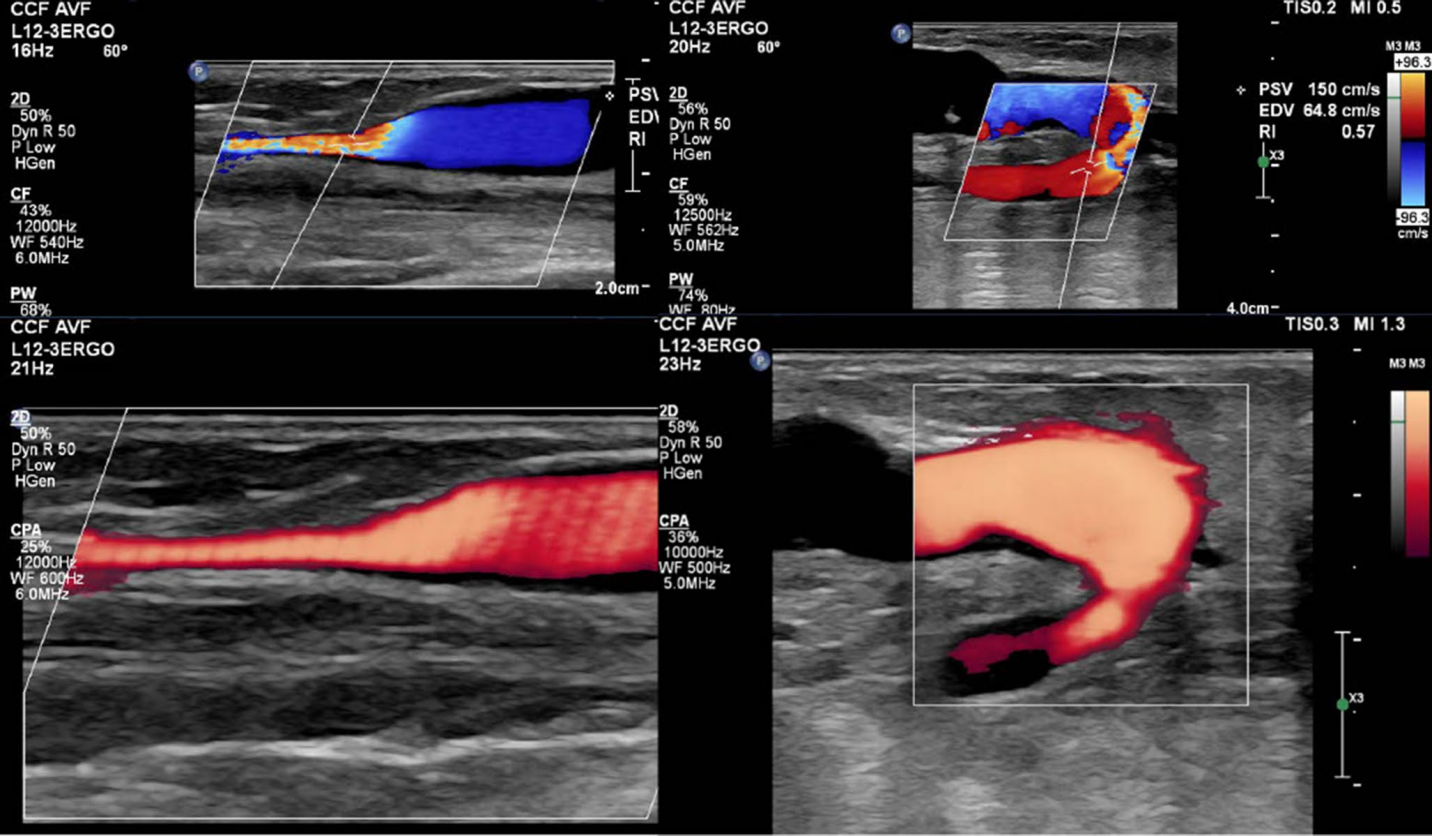
*Left*: Arterial Doppler Ultrasound (DUS) showing 50–99% stenosis of proximal ulnar artery in patient with Ulnar-Basilic Arterio-Venous Fistula. *Right*: Arterial DUS showing 50–99% stenosis of the arterial anastomotic site of Ulnar-Basilic Arterio-Venous Fistula

**Fig. 3:**
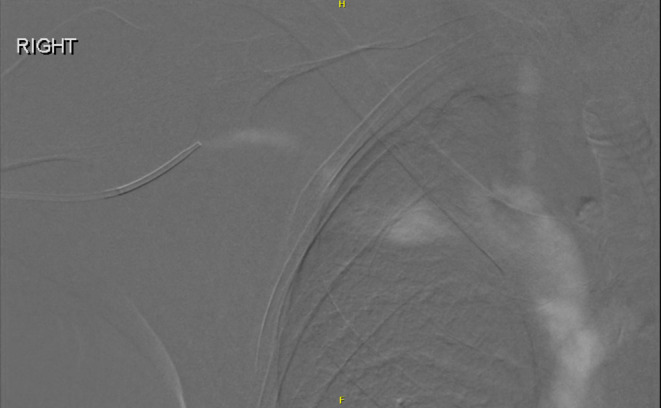
CO2 Venography showing patent right cephalic outflow vein and central venous system with no evidence of stenosis

**Fig. 4:**
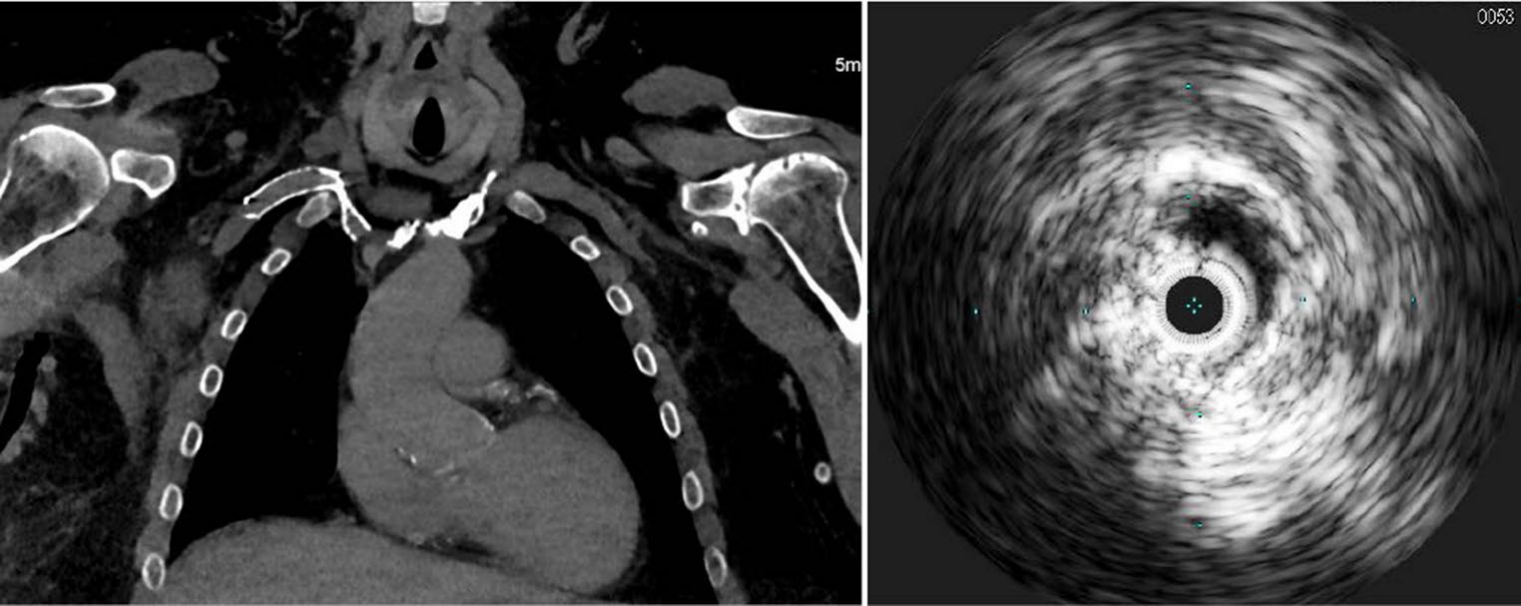
*Left*: Computed Tomography scan showing compression of the stent extending from the subclavian vein and terminating immediately above the confluence of the brachiocephalic veins as it courses over the first rib with accentuation in the arms down position with apparent occlusion. *Right*: Intra-vascular Ultrasound showing stent compression

The increased amount of native AVF creation in the “fistula first” era has resulted in an unintended increase in the occurrence of AVF non-maturation, which underscores the need for improved selection criteria based on pre-access imaging [17]. Lok et al. devised a clinical prediction model to estimate the risk of fistula non-maturation. Subsequently, certain patient groups have been identified to have reduced rates of AVF maturation and patency [18]. Those groups include patients with a history of diabetes or stroke, small or calcific arterial and venous anatomy, obesity, female sex, advanced age, and frailty [19–21]. Preoperative DUS may reliably identify these factors. Primary AVG or staged AVG, with the creation of conditioning fistula first, may be more appropriate in these patients [22, 23] (See Figs. 1, 2, 3 and 4).

### Guidelines

Kidney Disease Outcomes Quality Initiative (KDOQI), the most recent guideline for vascular access published in 2019 by Lok et al., strongly emphasizes preoperative clinical examination to assess patients and their vessels for AV access creation [24]. KDOQI *suggests* pre-access DUS in selected patients rather than routine vascular mapping in all patients. The guideline suggests the use of DUS in patients with a high risk of AVF failure, risk or history of peripheral vessel damage and central venous stenosis, and if physical examination is limited. However, the European Society of Vascular Surgery (ESVS) recommends pre-access DUS in all patients [24]. An RCT by Smith et al. found no significant difference in primary failure rates and complications between the selective and routine DUS imaging groups [25].

### Criteria of the inflow arterial system

DUS B-mode imaging is important to assess the diameter, caliber, and degree of atherosclerotic disease, including the presence of calcium within the inflow arterial system [26, 27]. These findings are paramount due to their correlation with improved AVF outcomes [27, 28]. The average diameter of the radial artery ranges from 2 to 3.5 mm. Broad variability has been reported in the recommended diameter parameters, which hinders the ability to make recommendations for the minimal lumen size. KDOQI now proposes no minimum diameter but careful evaluation for vessels < 2 mm in diameter [29]. A study found that a radial artery diameter of less than 1.6 mm was associated with early AVF failure, whereas other studies reported this threshold to be as high as 1.9 mm [14, 30–32]. Silva et al. proposed a criteria of arterial lumen diameter of 2.0 mm or more, which was confirmed by others and therefore served as the basis of KDOQI’s recommendation in 2006 [27, 33, 34]. However, some studies argue that arterial diameter is not a reliable predictor of AVF success and suggest AVF creation even for diameters less than 2.0 mm [35].

Yerdel et al. reported that an arterial flow value of ≥ 40 ml/min preoperatively was associated with increased fistula flow rates [36]. Sedlacek evaluated the Peak systolic velocity (PSV) as a predictor of AVF success, and a 50 cm/s PSV threshold was proposed for AVF success; however, other investigators found no significant correlation between PSV and successful AVF maturation [33, 37]. The hyperemic resistive index (RI) measures an artery’s ability to dilate postoperatively. A study reports that RI < 0.7 was associated with 95.3% successful AVF, whereas RI > 0.7 decreased the success rate to 38.7% [14, 31]. KDOQI does not recommend using flow rates, PSV, and RI for pre-access planning because if the threshold for arterial luminal diameter is met, it renders PSV and flow rates irrelevant [38].

DUS assists in obtaining information regarding arterial morphologic parameters, such as intimal hyperplasia, medial fibrosis, and vascular calcification which are consequential in AVF outcomes [39]. Various pilot studies have reported a positive correlation between intimal hyperplasia and AVF non-maturation [40, 41]. Advanced age and diabetes mellitus were found to be strongly associated with arterial medial fibrosis. Medical fibrosis is proposed to cause increased vascular stiffness and acts as a limiting factor in AVF maturation [39]. In a five-year follow-up study, vascular calcification severity is correlated with worse AVF patency [42]. However, histological micro-calcification was not found to be predictive of AVF non-maturation [43]. Diabetes is associated with medial calcinosis leading to high resistance distal to the dialysis access and thus increasing the risk of steal syndrome [44, 45].

### Criteria of the venous outflow system

Mendez et al. reported significantly higher maturation of the AVF with greater than 2.0 mm preoperative cephalic vein diameter [46]. A study including a receiver operator curve analysis reported 2.6 mm as the forearm cephalic vein diameter cutoff for women to ensure successful AVF maturation, whereas no such cutoff point was found in men [47]. The proposed threshold of 2.5 mm for AVF and 4.0 mm for AVG by Silva et al. are commonly applied [27]. KDOQI previously suggested a vein diameter of 2.5 mm, though current KDOQI recommendations propose no minimum threshold and suggest caution for a vein diameter of < 2 mm [29, 34].

Additional anatomic factors such as vessel wall quality and vessel depth are important preoperative factors. Vessel depth and the need for superficialization or transposition are independent factors for fistula dysfunction and cannulation ease [48, 49]. Large body habitus is more likely to leave insufficient length for cannulation and may necessitate conversion to a bridge prosthetic graft [50]. Finally, new anatomic factors are currently developed for the evaluation of candidacy for percutaneous fistula options [17].

AVF creation with an endovascular AVF system necessitates the DUS vascular mapping, whether Ellipsys (Avenu Medical, California) or WavelinQ (Becton Dickinson, New Jersey) devices are used [51]. The anatomical considerations for endovascular AVF include the presence of a perforating vein with a diameter greater than 2 mm connecting the deep and superficial venous system with a flow direction from the deep to the superficial venous system, inflow artery, and target vein with a minimum of 2 mm and 2.5 mm diameter, respectively and absence of thrombosis or stenosis as assessed by DUS [52, 53]. Ellipsys has an additional criterion of < 1.5 mm distance between the perforator vein and proximal radial artery [54]. The FDA’s proposed device-specific vascular eligibility parameters must also be met for both devices [52].

### Advantages/disadvantages of the modality

DUS offers an inexpensive and non-invasive tool for pre-access surgical planning. Being readily available and portable, DUS is an essential diagnostic imaging technique. It also reduces exposure to nephrotoxic contrast agents and ionizing radiation. However, the use of DUS has its limitations. DUS is an operator-dependent modality and has a limited field of view with inter-reader variability. DUS, being a 2D imaging modality, looks at 3D targets and, therefore, relies on the operator’s interpretation of the anatomy. Due to overlying bony structures, evaluation of the central venous system and associated stenotic disease is limited with DUS [26, 55] Table 1.

**Table 1:** Imaging modalities utilized in pre-access planning of surgical vascular access, along with their advantages, disadvantages, and the guidelines from KDOQI and ESVS regarding their application

	Advantages	Disadvantages	KDOQI guidelines	ESVS guidelines
DUS	- Non-invasive - No Ionizing Radiations - No nephrotoxic contrast - Widely available - Portable - Inexpensive	- Operator dependence - Interobserver variability - limited field of view - Two-dimensional modality - Inability to visualize the central venous system - Small field of view, 2D	Suggests selective preoperative ultrasound in patients at high risk of AV access failure **(Conditional Recommendation**,** Low Quality of Evidence)**	Pre-operative ultrasonography of bilateral upper extremity arteries and veins is recommended in all patients when planning the creation of vascular access **(I**,** A)**
DSA or DSV	- Gold standard modality - Ability to diagnose and intervene in a single procedure - Visualizes central venous system - Ability to create Endovascular-AVF - Real-time observation	- Invasive - Radiation exposure - Contrast exposure - Interobserver variability - Expensive - limited spatial resolution - Prone to artifacts - Small field of view, 2D	It is reasonable to use venography for suspected central vein occlusion while considering the patient’s clinical circumstances and residual kidney function. **(Expert Opinion)**	There are no recommendations for preoperative use
MRA	- Provides hemodynamic information - Visualizes central venous system - Evaluate extravascular structures - Multiplanar imaging - Large field of view, 3D - Minimal Inter-observer variability	- Risk of nephrogenic systemic fibrosis with gadolinium contrast - Increased exam time - Expensive - Artifacts	There are no recommendations for preoperative use	Contrast-enhanced MRA is not recommended in patients with end-stage renal disease. **(III**,** C)**
CTA	- Visualizes central venous system - Evaluate extravascular structures - High-resolution, multiplanar imaging - Large field of view, 3D - Relatively decreased examination time - Minimal Inter-observer variability - Multiplanar reconstruction possible	- Radiation exposure - Contrast exposure - Artifacts - Limited Soft Tissue Contrast	There are no recommendations for preoperative use	CTA may be considered in patients with inconclusive ultrasonographic or angiographic results concerning the degree of central venous stenosis. **(IIb**,** C)**

### Comparison with clinical examination

The efficacy of DUS was determined by comparing a group of patients with pre-access imaging to a group with a clinical examination (CE) alone. The group with pre-access imaging showed improved patency rates in the immediate postoperative period and increased mean duration of primary patency (14.7 ± 16.8 vs. 11.9 ± 9.4 months) compared to CE alone [56]. Gyori et al. compared DUS to examination alone for pre-access planning and analyzed long-term outcomes and cost-effectiveness. The DUS group showed improved primary patency rates (62% vs. 26%, *p* <.05), reduced rates of revision (25.4% vs. 59.4%, *p* <.0001), reduced revisions per patient over a median follow-up of 24.5 months (0.36 ± 0.71 vs. 1.06 ± 1.55, *p* <.0001) and decreased overall cost per patient, underscoring the benefit of ultrasound in planning of the dialysis circuit vascular access [57]. A meta-analysis in 2015 echoed similar findings with decreased odds of immediate AVF failure when performing DUS for presurgical planning [58]. However, the benefit of DUS over clinical examination remains a matter of debate, with two RCTs and one observational study showing no statistically significant difference in AVF primary failure, primary patency, frequency of secondary interventions, or mortality when comparing DUS and CE [59–61].

### Comparison with other imaging modalities

Various studies reported DUS in comparison with other imaging modalities and CE alone. Stoumpos et al. found Ferumoxytol magnetic resonance angiography (MRA) to be more predictive of the outcome of arteriovenous fistula surgery. However, it needs to be emphasized that this approach is less cost-effective than DUS, and in addition, Ferumoxytol is not FDA-approved as an MRA contrast agent [62]. A study comparing digital subtraction angiography (DSA) and DUS in the postoperative period found DUS to have comparable accuracy [63]. Baz et al. compared Multi-slice Computed Tomography Angiography (CTA) with DUS in the postoperative period and found comparable results between the two when DUS is operated by an experienced hand, however CTA was superior for evaluation of the central venous system [64]. Despite its limitations, DUS provides an economical imaging tool with high diagnostic accuracy and predictability of AVF patency and therefore serves as the modality of choice for the surveillance of AVFs [14, 65].

### Future developments

Endothelial reactivity is the ability of the vessel wall to respond to stress, and its assessment obtained through DUS may have important implications. Chronic kidney disease (CKD) is associated with variation in endothelial reactivity and may be a potential predictor of AVF maturation [66]. Contrast-enhanced DUS use is reported to have superior flow detection and allows appropriate stenosis detection and thrombus delineation [67]. Velocity Vector Imaging, a novel US analysis technique, assesses longitudinal and radial tissue motion associated with plaque burden in carotid atherosclerotic disease. This technique may provide the basis for future techniques in assessing AVF maturation [26, 68, 69]. Wang et al. developed an automated sonographic power law model that accurately describes blood flow patterns in the upper extremity and may be helpful in the future to effectively identify flow disturbances that may indicate stenotic disease or inadequate vessel wall remodeling, thereby potentially enabling a timely intervention [70].

## Digital subtraction angiography (DSA)

### Technical background

The introduction of the Seldinger technique in 1953 enabled widespread adaptation of diagnostic angiography as a safe imaging modality [71]. For many years, DSA has been the gold standard for visualizing arterial anatomy prior to AVF creation. The DSA technique involves acquiring a digital fluoroscopic image of the target area as a reference to subtract the extra-vascular static tissues after contrast has been injected. This allows the vessels filled with contrast to appear darker and more enhanced on a lighter (“zeroed out”) background [72].

### Clinical applications of the modality

DSA can be used in vascular access planning to adequately assess the inflow arterial segments of the future AVF and it is particularly useful for delineating the central arterial anatomy including identifying stenotic disease [26]. Asif et al. reported the presence of access inflow stenosis in 35% of the cases with clinical evidence of vascular access stenosis or thrombosis [73]. This study underscores the importance of DSA in imaging the arterial inflow segments related to central arterial pathologies, which cannot be visualized directly by DUS [74]. However, the presence or absence of central arterial lesions can be indirectly identified through preoperative mapping with DUS, which measures the bilateral brachial artery pressures with a pressure differential of more than 20 mm Hg indicative of subclavian, innominate, or axillary artery stenosis. However, the exact location of the central arterial stenosis cannot be identified with DUS. Arterial duplex can usually see the origin of the right subclavian artery and indirectly tardus parvus or turbulent waveform pattern in the presence of a left subclavian artery stenosis. Hence, KDOQI and ESVS do not recommend regular use of DSA for pre-access imaging [24, 29]. Postoperatively, fistulogram, a technique following the same principle as DSA, is routinely used if non-maturation or stenosis of the fistula is suspected leading to fistula malfunction.

Doelman et al. compared the ability of DUS, MRA, and DSA to detect stenosis in the vascular access circuit. They reported that DSA demonstrates superior sensitivity, specificity, PPV, and NPV and can diagnose all hemodynamically significant stenosis. However, they suggested using DUS as the initial imaging modality due to its noninvasive nature, with DSA to be performed for confirmation of stenotic disease along with concomitant endovascular treatment [75].

### Advantages/isadvantages of the modality

DSA is the gold standard for evaluating the arterial system. It has superior sensitivity and specificity for visualizing the arterial system and enables endovascular treatment in the same session [55]. The technique can also guide the endovascular-AVF creation [76] Table 1.

The routine use of DSA is limited by the exposure to radiation and contrast administration, along with the cost, availability, and time needed for patient preparation. The administration of contrast does not pose a problem in ESRD patients without residual renal function. However, in CKD patients and post-renal transplant patients with residual renal function, nephrotoxic iodine-based contrast agents should be avoided. In these patients an alternative to avoid contrast-related worsening of renal function is using carbon dioxide (CO2) as contrast media [77]. DSA is also a primarily intraluminal technique with a very limited ability to evaluate vascular walls and extra-vascular structures [26, 55]. DSA for evaluation of the central arterial vasculature is usually performed from a brachial or femoral access approach. Although uncommon, arterial vascular access carries the risk of various access site complications, such as pseudoaneurysm formation [78].

## Digital subtraction venography (DSV)

### Technical background

Thomas and Andress initially described the Venography of the upper extremity in 1971 [79]. Digital subtraction venography is the gold-standard imaging modality for assessing the outflow venous system, notably for visualizing the central venous system in pre-access planning [80]. DSA follows a similar technique as DSA described beforehand, and thus, the technique encounters similar technical advantages and disadvantages. As described by Surratt et al., the high incidence of central venous stenosis associated with temporary CVC provides an impetus for appropriate assessment of the central venous system in these patients [81]. The assessment of central veins is also critical due to the high incidence of failed dialysis circuit vascular access creations with subsequent recirculation phenomenon and increased bleeding rates in patients with central venous stenosis [82].

### Clinical applications of the modality

Patients presenting with prominent venous collaterals, upper extremity swelling, a prior history of CVC, and multiple prior accesses in the same extremity should be suspected of having central venous stenosis. Therefore, evaluation of the central venous system with DSV is of the utmost importance in these patients [83]. KDOQI recommends using DSV with clinical correlation in pre-access planning for patients suspected of having central venous stenosis [29]. DSV is also helpful in patients with a history of prior ipsilateral vascular access procedures since US vein mapping diminishes sensitivity in these cases. DSV remains the gold standard for assessing venous thrombus and obstruction in the post-operative setting. It also provides an option to intervene during the same procedural session [84]. Elsharawy et al. gauged the impact of venography on pre-access planning and reported improved success rates by selecting more suitable veins and increasing the overall number of dialysis accesses created [85]. The anatomical criterion for successful AVF creation in DSV is similar to DUS: a vein diameter of at least 2.5 mm and a 6 cm long straight cannulation segment [86]. However, as previously mentioned most recent KDOQI guidelines do not provide a minimum cutoff value for arterial and venous diameters [29].

### Comparison with other imaging modalities

Passman et al. compared DSV and DUS for the detection of upper extremity venous outflow obstruction and reported that DSV has superior diagnostic ability [87]. A study comparing venography and contrast-enhanced 3D MR venography showed consistency of results between the two modalities [88].

### O2 venography

CO2 venography provides a viable and safe alternative to conventional iodine contrast-based venography [89]. Its use in assessing upper limb vein patency and stenosis has demonstrated an 85% sensitivity, 97% specificity, and 95% accuracy [90]. Heye et al. demonstrated the value of CO2 venography in venous mapping for presurgical dialysis circuit vascular access planning. AVF creation based on CO2 venography resulted in an overall maturation rate of 84% and one-year primary and secondary patency rates of 63% and 71%, respectively [91]. KDOQI recommends using CO2 venography in patients with CKD to preserve residual renal function [29]. Although adverse effects with this technique are rare, it may lead to neurotoxicity, air contamination of the delivery system, cardiac events, and vapor lock in the pulmonary artery, causing hypoxia and hypotension [92]. Therefore, it should be only used if there is a clear clinical indication.

## Magnetic resonance angiography (MRA)

### Technical background

MRA encompasses several techniques based on magnetic resonance imaging (MRI) to study arterial and venous vasculature. Though DSA and DSV are the gold standard, MRA provides a viable, non-invasive alternative to adequately visualize the vasculature without exposure to ionizing radiation [55, 93]. Different MRI techniques, such as contrast-enhanced MR Angiography or non-contrast MR angiography, such as the quiescent interval slice selective (QISS) sequence, can be employed to visualize the arterial and venous vasculature for pre-access planning [26, 94].

Doelman et al. reported that the contrast-enhanced MRA has a 96% sensitivity, 98% specificity, 94% positive predictive value (PPV), and 98% negative predictive value (NPV) for stenosis detection in failing VAC [75]. A pilot study by Bode et al. compared contrast-enhanced and non-contrast MRA images to assess upper extremity and central vasculature and reported no significant differences in image quality and suggested the use of non-contrast MRA as a viable alternative in certain patient populations, such as those with ESRD [95]. The off-label use of Ferumoxytol (Feraheme, AMAG Pharmaceuticals, Waltham, MA) as an alternative in MRA is reported to be safe in ESRD patients with a lower rate of adverse events and anaphylaxis [96–98].

### Clinical applications of the modality

MRA may be useful clinically in patients with concern for central venous stenosis and a prior history of multiple central venous access [38]. MRA may be more suitable over CT or DSA in patients with residual renal function [95, 96, 99]. Planken et al. reported MRA as a more accurate modality to determine upper extremity venous diameters than DUS, and MRA enables accurate detection of upper limb arterial and venous stenosis for presurgical vascular access planning [100]. Non-contrast MRA led to accurate and reliable detection of stenosis in **≥** 50% of cases in dysfunctional AVF [101]. MRI also provides an option to obtain hemodynamical information via 4D flow, including parameters such as flow, pressure, and wall shear stress. Some hemodynamic parameters were demonstrated to be closely related to AVF maturation and complications [102].

### Advantages/disadvantages of the modality

The advantages of MRA include the lack of radiation exposure, the non-invasive technique, and the modality providing multi-planar anatomical images with superior spatial resolution and a high contrast-to-noise ratio [55]. Non-contrast MRA with diagnostic image quality is very adventitious in the patient population requiring AVF with remaining renal function [95]. That being said, newer Gadolinium-based contrast agents are considered safe in patients with CKD since Gadolinium is not nephrotoxic, and the risk of nephrogenic systemic fibrosis with the newer contrast agents is negligible. Unlike DUS, the imaging capabilities of MRI are not limited by bony structures, and therefore, MRA may be helpful in evaluating the central venous system [26]. Unlike DUS, MRA has minimal interobserver variability. The primary limitations of this modality are its susceptibility to artifacts, limited availability, high associated costs, and longer examination time [26, 55] Table 1.

### Comparison with other imaging modalities

Magnetic Resonance venography (MRV) was compared to conventional venography by Menegazzo et al., who reported a good correlation between diameter measurement overall and on a vein-to-vein basis. Presurgical MRV also demonstrated an increased number of patent veins and a better correlation with intraoperative findings [103]. Another study comparing time-of-flight magnetic resonance angiography to DSA revealed 83.3% congruence [104]. Compared to DUS, MRA was reported to be a more accurate predictor of upper extremity venous diameter [100]. Froger et al. compared MRA and DSA to determine the ability to detect dysfunctional AVF and reported high sensitivity and specificity for MRA compared to DSA. However, unlike DSA, the clinical implications of MRA are limited due to the inability to perform concurrent interventions [105]. A similar study comparing DUS, MRA, and DSA concluded that DUS should be used as the initial imaging modality of choice to detect dysfunctional AVF, DSA to detect stenoses eligible for endovascular intervention with concomitant intervention, and MRA only if DSA is inconclusive [75].

## Computed tomography (CT) scan

### Technical background

Multidetector computed tomography (MDCT) angiography provides high-resolution spatial imaging using ionizing radiation in a very short acquisition time [106]. It is a valuable technique for evaluating arterial and venous vasculature for pre-access planning and detection of stenotic disease in the postoperative setting [107]. MDCT angiography is reported to have a 98.7% sensitivity, 97.5% specificity, 98.8% PPV, 97.2% NPV, 98.3% accuracy, and high interobserver correlation in evaluating failing AVFs [107]. The 3-D reconstructions and Maximum Intensity Projection (MIP) images produced by MDCT angiography offer freely rotatable projection angiogram-like images to allow proper visualization of vascular lesions. MDCT angiography requires nephrotoxic contrast administration for adequate assessment, which is particularly problematic for CKD patients with residual renal function who require AVF creation for anticipated hemodialysis initiation in the near future [93].

### Clinical applications of the modality

MDCT angiography can be used for central venous system assessment and accurately demonstrates extrinsic mass compression, which may not be readily visible with DUS. CT can also be helpful in identifying subclavian vein compressions around the costoclavicular junction, a significant finding in patients with future AVF requirements [26, 106]. Heye et al. found MDCT angiography to be comparable to DSA, reproducible, and reliable as an imaging technique for stenosis or occlusion detection in patients with AVF for hemodialysis [108]. On DSA, non-concentric stenosis may be missed secondary to its 2D imaging capabilities, though CT angiography provides 3D information, hence facilitating the detection of these stenotic lesions. Although there is a paucity of studies looking at MDCT angiography as an imaging modality for pre-access venous mapping, this non-invasive 3D angiography technique, with MIP and volume rendering, can be used in selective cases [109, 110]. Intraprocedural cone-beam CT is another technique that can obtain limited cross-sectional information, thereby providing a real-time vascular road map in complex AVF procedures [111].

### Advantages/disadvantages of the modality

CT imaging is recognized for its high spatial resolution and provides significant advantages from a practical standpoint as a non-invasive modality with 3-dimensional imaging capabilities, a large field of view, and the modality is not operator-dependent. CT provides superior anatomical delineation, and the examination time is usually less than one minute with in-room time of fewer than 10 min [55]. This technique’s limitations include exposure to ionizing radiations, though newer technological developments such as dual source CT have the potential to significantly decrease radiation exposure [26]. The administration of nephrotoxic contrast agents is problematic for CKD patients with residual renal function, as discussed previously Table 1.

### Comparison with other imaging modalities

Ko et al. compared MDCT angiography and DSA. No difference was found in the detection and grading of stenosis at various anatomical segments [107, 108]. No discrepancy was found between MDCT angiography and intra-operative findings. Another study reported that MDCT performed superiorly to DUS in detecting subclavian vein occlusion, SVC occlusion, chest wall venous collateralization, and innominate vein occlusion [64]. There is a paucity of data comparing MDCT and MRA regarding presurgical dialysis circuit vascular access planning and future studies in this are warranted.

## Intravascular ultrasound (IVUS)

IVUS is an evolving technology that has improved post-procedural outcomes in percutaneous coronary intervention, and evidence to support its benefits in peripheral vascular intervention is steadily increasing [112]. A pilot study by Ross et al. comparing IVUS and DSV in patients with failing hemodialysis access grafts revealed that IVUS altered the treatment plan in 76% of the patients, and 80% of patients in the IVUS group remained free from graft abandonment. They concluded that the addition of IVUS to standard venography holds the potential to extend the time to the first reintervention [113]. Another study identifying central venous lesions in hemodialysis patients revealed that IVUS has a superior ability to identify significant aspects of a central venous outflow obstruction compared to DSA [114]. IVUS is also valuable in the detection of venous thoracic outlet syndrome, and Kim et al. suggested that IVUS was more sensitive for the detection of central vein stenosis compared to 2D venography, given the dynamic nature of the lesion morphology [115]. In selected ESRD patients, IVUS may be beneficial for accurate assessment of the venous geometry, taking the pliability of the central venous system into consideration [116].

## Conclusion

Hemodialysis is the mainstay therapy for ESRD, and it requires the patency and maturation of a dialysis access circuit. Pre-access imaging can be pivotal in determining and predicting access outcomes and guiding patient management. Following KDOQI guidelines, a combination of a patient’s medical history, physical examination, and DUS is a widely accepted standard practice in presurgical vascular access planning, with other imaging modalities reserved for selected patients. However, with evolving MDCT technology, contrast and non-contrast MRA options, and the increasing availability of IVUS, the diagnostic accuracy, choice of imaging, and procedural outcomes are bound to improve in the future, along with a more integrated approach to gathering imaging information to optimize outcomes in the ESRD population requiring functional vascular access for hemodialysis.

## Data Availability

No datasets were generated or analysed during the current study.
